# Airway smooth muscle relaxation results from a reduction in the frequency of Ca^2+ ^oscillations induced by a cAMP-mediated inhibition of the IP_3 _receptor

**DOI:** 10.1186/1465-9921-7-34

**Published:** 2006-02-23

**Authors:** Yan Bai, Michael J Sanderson

**Affiliations:** 1 Department of Physiology, University of Massachusetts Medical School, 55 Lake Avenue North, Worcester, MA 01655, USA

## Abstract

**Background:**

It has been shown that the contractile state of airway smooth muscle cells (SMCs) in response to agonists is determined by the frequency of Ca^2+ ^oscillations occurring within the SMCs. Therefore, we hypothesized that the relaxation of airway SMCs induced by agents that increase cAMP results from the down-regulation or slowing of the frequency of the Ca^2+ ^oscillations.

**Methods:**

The effects of isoproterenol (ISO), forskolin (FSK) and 8-bromo-cAMP on the relaxation and Ca^2+ ^signaling of airway SMCs contracted with methacholine (MCh) was investigated in murine lung slices with phase-contrast and laser scanning microscopy.

**Results:**

All three cAMP-elevating agents simultaneously induced a reduction in the frequency of Ca^2+ ^oscillations within the SMCs and the relaxation of contracted airways. The decrease in the Ca^2+ ^oscillation frequency correlated with the extent of airway relaxation and was concentration-dependent. The mechanism by which cAMP reduced the frequency of the Ca^2+ ^oscillations was investigated. Elevated cAMP did not affect the re-filling rate of the internal Ca^2+ ^stores after emptying by repetitive exposure to 20 mM caffeine. Neither did elevated cAMP limit the Ca^2+ ^available to stimulate contraction because an elevation of intracellular Ca^2+ ^concentration induced by exposure to a Ca^2+ ^ionophore (ionomycin) or by photolysis of caged-Ca^2+ ^did not reverse the effect of cAMP. Similar results were obtained with iberiotoxin, a blocker of Ca^2+^-activated K^+ ^channels, which would be expected to increase Ca^2+ ^influx and contraction. By contrast, the photolysis of caged-IP_3 _in the presence of agonist, to further elevate the intracellular IP_3 _concentration, reversed the slowing of the frequency of the Ca^2+ ^oscillations and relaxation of the airway induced by FSK. This result implied that the sensitivity of the IP_3_R to IP_3 _was reduced by FSK and this was supported by the reduced ability of IP_3 _to release Ca^2+ ^in SMCs in the presence of FSK.

**Conclusion:**

These results indicate that the relaxant effect of cAMP-elevating agents on airway SMCs is achieved by decreasing the Ca^2+ ^oscillation frequency by reducing internal Ca^2+ ^release through IP_3 _receptors.

## Introduction

A major symptom of asthma is an excessive contraction of airway smooth muscle cells (SMCs) which results in airway hyper-reactivity. To alleviate this acute and chronic airway constriction, β-adrenergic agonists, that relax SMCs, are commonly administered [1]. Yet, despite their efficacy and wide-spread application, the signaling pathways underlying the relaxing effect of β-agonists are not fully understood.

It is generally accepted that stimulation of β-adrenergic receptors activates adenylyl cyclase (AC), via receptor associated G proteins, to increase cAMP levels to mediate SMC relaxation [2]. The role of cAMP in relaxation has been confirmed with other compounds that increase intracellular cAMP concentration ([cAMP]_i_), such as forskolin (FSK) that directly activates AC, theophylline that inhibits phosphodiesterase and 8-bromo-cAMP that is an analog of cAMP [3-5]. The traditional mechanism for cAMP action is via the stimulation of protein kinase A (PKA) to phosphorylate a variety of target proteins to induce airway SMCs relaxation. Alternatively, cAMP may act independently of PKA by interacting with exchange proteins (EPACs) [6]. EPACs have been found to activate PCLε to enhance IP_3 _induced Ca^2+ ^release in non-smooth muscle cells [7]. However, before the specific details of cAMP-mediated signaling can be explored, it is essential to initially identify the fundamental action of cAMP on the mechanisms that regulate SMC contraction.

Airway SMC contraction is determined by the balance between phosphorylation and de-phosphorylation of the regulatory light chain of myosin (rMLC). Phosphorylation of rMLC is induced by Ca^2+^-calmodulin activated myosin light-chain kinase (MLCK). De-phosphorylation of rMLC is believed to be mediated by myosin light chain phosphatase (MLCP) [8]. As a result, SMC relaxation may be the result of different cellular pathways that culminate in either or both a reduction in MLCK activity and an increase in MLCP activity.

Perhaps the most direct method of relaxation is a reversal of the Ca^2+ ^response that stimulates MLCK activity and contraction. Ca^2+ ^signaling in airway SMCs is frequently induced by agonists such as ACh, 5-HT and ATP and consists of Ca^2+ ^oscillations [9-11]. In our recent studies with lung slices, we found that the frequency of these Ca^2+ ^oscillations correlated with the contractile state of the airways, a relationship that indicates the SMC tone is regulated in a frequency-modulated manner [12-14].

In previous studies with isolated tracheal SMCs, cAMP was found to modulate the frequency of Ca^2+ ^oscillations [15,16]. However, its effect on airway contraction or the mechanism of action was not determined. In other studies with different SMC types, it has been suggested that cAMP interacts with intracellular Ca^2+ ^signaling pathways at multiple sites [17]. These include a decrease in Ca^2+ ^influx [3,15,16,18], particularly by the activation of large conductance Ca^2+^-activated K^+ ^channels (BK_Ca_) to hyperpolarize the membrane [19-21], an increase in Ca^2+ ^efflux [22] or uptake by the sarcoplasmic reticulum [23,24], or an inhibition of agonist-induced IP_3 _formation [5,25]. All of these effects of cAMP would be expected to decrease the available intracellular Ca^2+ ^concentration ([Ca^2+^]_i_). However, it is unknown how any of these effects would influence the frequency of Ca^2+ ^oscillations of SMCs.

To explore the hypothesis that cAMP induces intrapulmonary airway relaxation by down-regulating Ca^2+ ^oscillations, we examined the effect of isoproterenol (ISO) and other cAMP elevating agents (FSK and 8-bromo-cAMP) on methacholine (MCh)-induced contractility and Ca^2+ ^signaling of airway SMCs in murine lung slices. The importance of using lung slices, instead of cultured or isolated cells, is that Ca^2+ ^signaling and airway contractility can be measured simultaneously. We found that cAMP induced relaxation of MCh-contracted airways and reduced the frequency of the Ca^2+ ^oscillations. In addition, we confirmed that cAMP did not affect Ca^2+ ^influx or the refilling of depleted Ca^2+ ^stores. By contrast, we found that an elevation of intracellular IP_3 _concentration ([IP_3_]_i_) reversed the effect of FSK by increasing the frequency of the Ca^2+ ^oscillations to induce contraction. In addition, we also show that cAMP inhibits the Ca^2+ ^release via the IP_3 _receptor. From these data, we suggest that airway relaxation induced by cAMP is achieved by decreasing the Ca^2+ ^oscillation frequency of airway SMCs by inhibiting IP_3_-induced Ca^2+ ^mobilization.

## Materials and methods

### Materials

Cell culture reagents were obtained from GIBCO/Invitrogen Corp. Oregon Green 488 BAPTA-1 AM and O-nitrophenyl EGTA AM (NP-EGTA AM) were obtained from Molecular Probes (Eugene, OR). Caged-iso-Ins(1,4,5)P_3_/PM was obtained from Alexis Biochemicals (San Diego, CA) and dissolved in DMSO, aliquoted and frozen at -80°C. Other reagents were obtained from Sigma-Aldrich or Calbiochem. Hanks' balanced salt solution was supplemented with 20 mM HEPES buffer (sHBSS) and adjusted to pH 7.4.

### Lung slices

The preparation of lung slices has been previously described in detail [14]. Briefly, male Balb/C inbred strain mice (Charles River Breeding Labs, Needham, MA, 7–10 weeks old) were killed by intra-peritoneal injection of 0.3 ml of pentobarbital sodium (Nembutal) as approved by the IACUC of the University of Massachusetts Medical School. The trachea was exposed and cannulated. After the thoracic cavity was opened, the lung lobes were slowly inflated with a warm agarose solution (2%, 37°C, ~1.3 ml). Subsequently ~0.2 ml of air was injected to flush the agarose out of the airway into the distal alveoli. The lungs were cooled at 4°C to gel the agarose. A single lung lobe was removed and sectioned with a vibratome (model EMS-4000; Electron Microscope Sciences, EMS) into slices of ~140 μm thick starting at the lung periphery. Airways were identified by their lining of epithelial cells with beating cilia. Serial sections were collected and transferred to culture solution containing DMEM supplemented with antibiotics and anti-mycotics and NaHCO_3_. Slices were maintained in culture media at 37°C and 10% CO_2 _for up to 3 days.

### Measurement of airway contraction and relaxation

Lung slices were observed in a custom-made perfusion chamber constructed from 2 cover-glasses sealed with thin strips of silicone grease (Valve lubricant and sealant, Dow Corning, Midland, MI) on a Plexiglas support. The slices were held in place with a small sheet of nylon mesh (CMN-300-B, Small Parts Inc, Miami Lakes, FL) with a small hole to view the selected airway and avoid the mesh from influencing the contraction of the airway. A gravity-driven perfusion system with a multi-tube manifold with a single output (Warner Instruments, Inc.) was used, in conjunction with valves (LFVA, Lee Company, CT) under TTL control, to exchange solutions in the chamber. The chamber volume was about 100 μl. The perfusion rate was 800 μl/min.

The lung slices were observed with an inverted microscope (IX71, Olympus) with a 10× objective and a zoom adapter. Phase-contrast images were recorded using a CCD camera (model LCL-902C, Watec America Corp, USA), a camera interface ("Picolo", Eurosys Inc., Belgium) and image acquisition software ("Video Savant"; IO industries, Inc, London, Ontario, Canada). Digital images were recorded in time-lapse (0.5 Hz) and analyzed with "NIH /Scion Image" software (Scion Corp.). The lumen area of the airway was measured by summing the number of pixels below a selected threshold grey level with respect to time. All experiments were performed at room temperature (RT, 20 ± 2°C).

### Measurements of [Ca^2+^]_i_

Slices were loaded for 30 minutes at 30°C with 20 μM Oregon Green 488 BAPTA-1 AM in sHBSS containing 0.1% pluronic (Pluronic F-127; Calbiochem) and 100 μM sulfobromophthalein. Lung slices were de-esterified for another 30 minutes at 30°C in sHBSS containing 100 μM sulfobromophthalein before being placed in the perfusion chamber as described above.

Fluorescence imaging was performed using a custom-built video-rate confocal or 2-photon scanning laser microscope. Either a 488 nm beam from a diode laser or an 800 nm beam from a Ti-sapphire laser (Tsunami, Spectra-Physics, Mountain View, CA) pumped with a 5 W 525 nm diode laser (Millennia, Spectra-Physics) was scanned across the specimen with two oscillating mirrors (X- and Y-scan) through an inverted microscope (Nikon DIAPHOT 200 for confocal microscopy; Olympus IX71FVSF-2 for 2-photon microscopy). For confocal microscopy, the emitted fluorescence (>510 nm) was separated from the excitation light by a dichroic mirror, a long pass filter and a confocal aperture [26]. For 2-photon microscopy, the emitted fluorescence was separated with a dichroic mirror (670uvdclp, Chroma Technology Corp, Rockingham, VT) and a long pass filter (E700SP or E600SP-2P, Chroma Technology Corp) positioned immediately below the objective. Emitted fluorescence was detected with a photomultiplier tube (PMT, R5929, Hamamatsu USA, Bridgewater, NJ). Grayscale images were recorded at 1, 15 or 30 Hz with a frame-grabber (Raven; Bit Flow, Inc). Changes in fluorescence intensity were obtained, frame-by-frame, from selected regions of interest (ROI, ~5 × 5 pixel). A line-scan analysis was made by extracting a row of pixels from each frame and aligning them in a time sequence.

### Flash photolysis of caged-Ca^2+ ^or caged-IP_3_

Flash photolysis of caged-Ca^2+ ^(NP-EGTA) or caged-IP_3 _was used to experimentally increase the [Ca^2+^]_i _or [IP_3_]_i_. Slices were initially loaded with Oregon Green 488 BAPTA-1 AM as described above. Subsequently, slices were incubated, at RT, with either 1 μM NP-EGTA AM (for 15 min) or 2 μM Caged-iso-Ins(1,4,5)P_3_/PM (for 1 hr) in sHBSS containing 0.1% pluronic and 100 μM sulfobromophthalein followed by de-esterification for 30 min in the sHBSS containing 100 μM sulfobromophthalein. The details of the flash photolysis setup have been previously described [27]. Briefly, a flash of UV light was produced from a mercury arc lamp with a mechanical shutter and focused into the microscope with a biconvex lens (focal distance 200 mm) through a bandpass filter (330 nm). The flash duration was determined by electronic control and the image acquisition software. The intensity of the flash was regulated by a neutral density filter (Optical Density, OD 0.5 ~ 1.0, transmission = log [1/OD]). The size of the area illuminated was adjusted with an iris diaphragm and measured by illuminating and monitoring the emitted fluorescence from a thin uniform layer of fura-2 salt solution (1 μM) between 2 cover glasses.

### Data analysis

Results are expressed as means ± S.E. Concentration-response curves of MCh, ISO and FSK were fitted using a non-linear interactive fitting program (Microcal Origin 7.0, Microcal Software Inc, MA, USA). Statistical significance was determined by using paired student's t-test.

## Results

**Figure 1:**
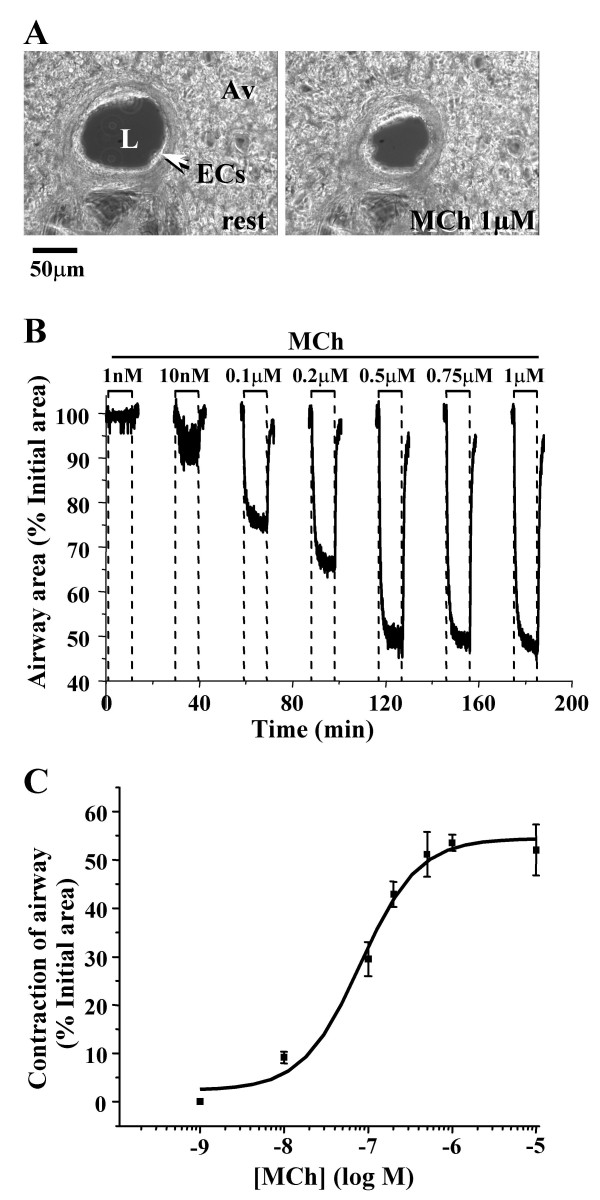
**The contractile response of airways in mouse lung slices to MCh. (A) **Low magnification phase-contrast images (scale bar = 50 μm) of the same airway before and 5 minutes after stimulation with 1 μM MCh. L, airway lumen; ECs, epithelial cells; Av, alveolar tissue. **(B) **The relative change in the area of the airway lumen (% of the initial area) in response to MCh from 1 nM to 1μM. During the interval between each application of MCh (15 min) the airway area was not recorded but the slice was continually washed with sHBSS. **(C) **The concentration-dependent contractile response of airways to MCh. Each point represents 5 to 7 experiments (mean ± S.E.) in different airways from at least 3 mice. The data were fitted with a logistic function.

Experiments were only performed on airways that were lined with epithelial cells (ECs) showing active ciliary beating and had a lumen that was free of agarose (Fig. 1A). To assess the contractile activity of the airway SMCs, the changes in the airway area were measured with time-lapse video microscopy and image-analysis. Upon exposure to MCh, at concentrations ≥ 100 nM, the airway quickly contracted. The luminal area was reduced to, and maintained at, a smaller stable size. Although lower concentrations of MCh (10 nM) induced a reduction in the airway lumen, this change was the result of twitching rather than a sustained contraction of the SMCs (Fig. 1B). The MCh-induced airway contraction was concentration-dependent over the range of 10 to 500 nM (Fig. 1C). A maximal contraction of about 52 % was achieved when the concentration of MCh was >0.5 μM. The EC_50 _was 78 nM.

### The relaxing effect of ISO on airways contracted by MCh

**Figure 2:**
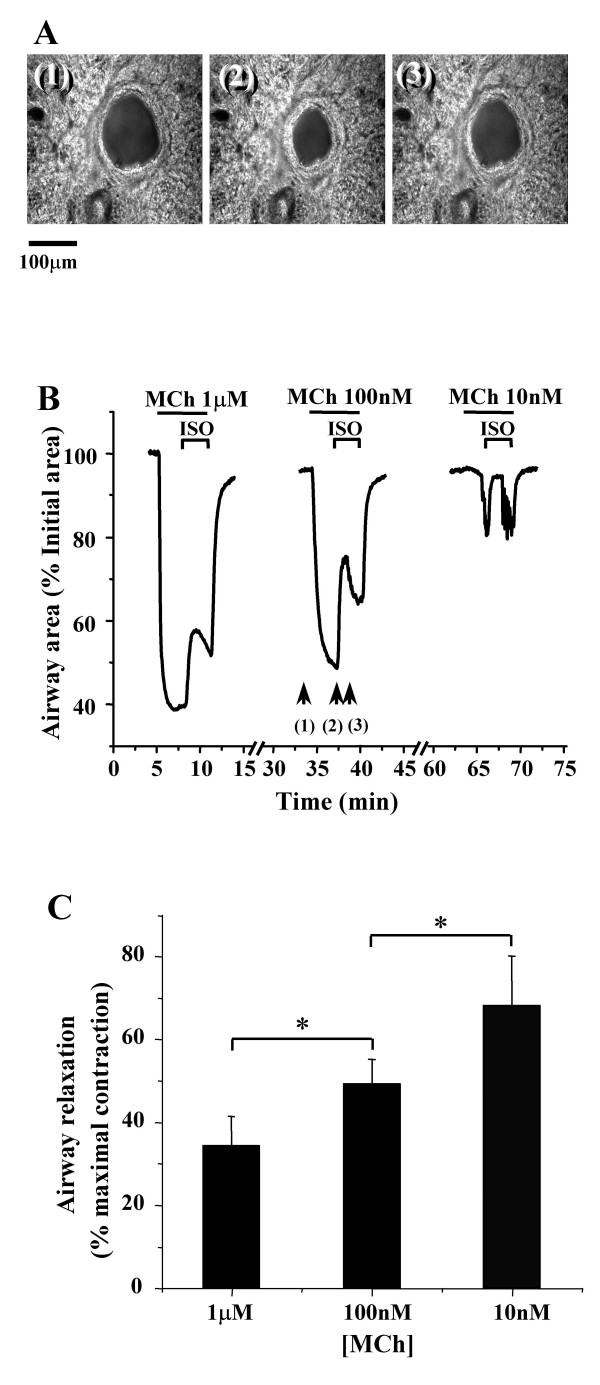
**Relaxation of contracted airways by ISO**. **(A) **A series of phase-contrast images of the same airway (scale bar = 100 μm) at times indicated by arrows in B (middle trace) showing (1) the initial state, (2) the contracted state induced by MCh (100 nM) and (3) the relaxed state induced by ISO (10 μM). **(B) **The changes in the area of an airway in response to ISO (10 μM) following contraction with MCh. The airway was contracted with decreasing concentrations of MCh (1μM to 10 nM) for 3 min and exposed to the same concentration of ISO (10 μM) for another 3 min. The slices were washed with sHBSS 15 minutes between each concentration of MCh. **(C) **Summary of the ISO-induced (10 μM) relaxation of airways contracted with different concentrations of MCh (n = 4 slices from 3 mice). The magnitude of the airway relaxation induced by ISO increased as the concentration of MCh was decreased (*, p < 0.05, comparison between two different MCh concentrations).

The relaxing effect of ISO on airways was established by initially contracting the airways with MCh for 3 minutes and subsequently exposing the airways to ISO in the presence of MCh. In the first set of experiments, the airways were contracted with a range of MCh concentrations (10 nM to 1 μM) and relaxed with a single concentration of ISO (10 μM). The increasing concentrations of MCh induced an increasing airway contraction and under each condition, addition of ISO induced airway relaxation during the first 2 minutes (Fig. 2A and 2B). This relaxation effect was not sustained even though the airways were continually perfused with MCh and ISO. After 2 minutes, the airways gradually re-contracted until the lung slice was washed with sHBSS at which time the airway fully relaxed. The magnitude of the initial relaxation, measured after 60 to 90 seconds of ISO exposure, decreased from ~68% to ~34% (% maximal contraction pre-exposed to ISO) as the concentrations of MCh increased from 10 nM to 1 μM (Fig. 2B and 2C). The relaxation response was significant different between MCh concentrations (n = 4 slices from 3 mice, p < 0.05). These results indicated that the efficacy of ISO at relaxing the airway is reduced by an increasing contractile stimulus.

Because MCh can stimulate the M_2 _receptor and this could possibly lead to the inhibition of adenylyl cyclase (AC) to enhance the contractile response, we examined the effects of the M_2 _receptor antagonist, methoctramine (0.2 μM) on ISO-induced relaxation. In the presence of the M_2 _antagonist, airway contraction induced by MCh (200 nM) was decreased by about ~5%. However, the transient relaxation induced by ISO (10 μM) was unchanged in the presence of methoctramine. A similar transient relaxation was also observed in response to ISO (10 μM), when the airway was pre-contracted with another agonist, serotonin (5-HT, 1 μM).

**Figure 3:**
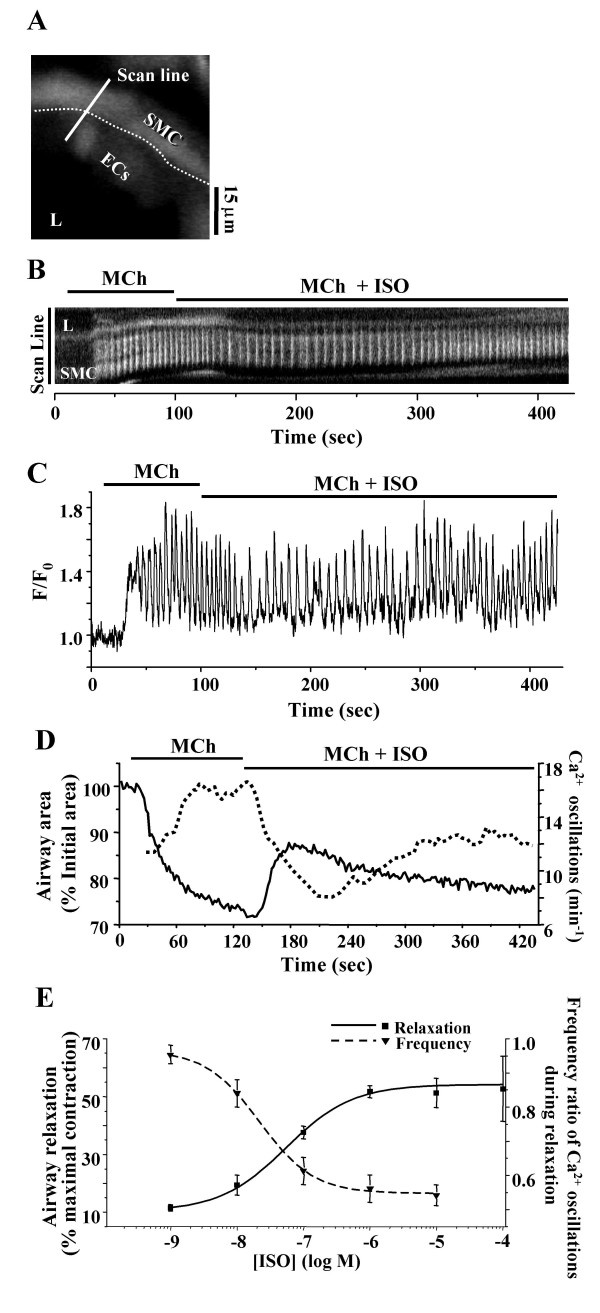
**The effect of ISO on the Ca^2+^signaling of airway SMCs. (A**) A fluorescence image of part of an airway obtained by two-photon microscopy under resting conditions. L, lumen; ECs, epithelial cells; SMC, smooth muscle cell. Dotted line indicates the interface between ECs and SMCs. Scale bar = 15 μm. The intracellular Ca^2+ ^signaling represented by **(B) **a line-scan plot, constructed from the white line across the SMC as indicated in **A**, and the sequence of recorded images and **(C) **a small ROI in the same airway SMC as shown in **B **in response to the MCh (200 nM) and ISO (1 μM). **(D) **The correlation of the Ca^2+ ^oscillation frequency (dotted line) and airway area (solid line) in a representative experiment with contraction induced by MCh (200 nM) and relaxation induced by ISO (10 μM). The Ca^2+ ^oscillation frequency was determined by the time interval between 2 adjacent peaks. The frequency of the Ca^2+ ^oscillations was inversely coupled to the contraction of the airway. **(E) **The mean concentration-dependent relaxation (solid line, ■) and the change in the frequency of the Ca^2+ ^oscillations (dash line, ▼) induced by ISO in airways contracted with MCh (200 nM). The mean Ca^2+ ^oscillation frequency after ISO exposure was expressed as a ratio to the Ca^2+ ^oscillation frequency during MCh exposure. As the concentration of ISO increased, the Ca^2+ ^oscillation frequency decreased. Each point represents 5 to 8 experiments (mean ± S.E.) in different airways from at least 3 mice for the contraction data and 5 – 6 experiments in different cells from at least 3 mice for the Ca^2+ ^oscillation data. Data were fitted with a logistic function.

In a second set of experiments, the airways were contracted with a single concentration of MCh and relaxed with different ISO concentrations. The previous data indicated that it was necessary to use a low MCh concentration in order that the relaxation responses could be differentiated. We determined that 200 nM MCh satisfied these requirements. In airways contracted with MCh (200 nM), ISO induced relaxation with a typical pattern of an initial relaxation followed by a slow re-contraction (Fig. 3D solid line). The magnitude of the initial relaxation was concentration-dependent from 1 nM to 1 μM ISO, ranging from 11 ± 1% (n = 5 slices from 3 mice) to 53 ± 2% (n = 6 slices from 4 mice) (Fig. 3E solid line). No further relaxation was induced by ISO at concentrations higher than 1μM (Fig. 3E).

### Ca^2+ ^events underlying airway contraction and relaxation

In response to 200 nM MCh, the airway SMCs demonstrated an initial increase of intracellular Ca^2+ ^which was followed by the onset of Ca^2+ ^oscillations (Figs. 3B and 3C). These Ca^2+ ^oscillations were superimposed on a slightly elevated baseline. The frequency of the Ca^2+ ^oscillations increased to a stable rate within 1 minute. Upon exposure to 1 μM ISO, the frequency of the Ca^2+ ^oscillations slowed, although the baseline of the Ca^2+ ^signal did not substantially change. In the continued exposure to ISO with MCh and after about 150 seconds, the frequency of Ca^2+ ^oscillations began to increase and approached a new steady rate; this rate was less than the initial rate induced by MCh alone.

The frequency of the Ca^2+ ^oscillations was determined from the period of each oscillation (time interval between 2 adjacent peaks of the Ca^2+ ^signal). As a result a correlation between the dynamic changes in the frequency of the Ca^2+ ^oscillations with the changes in the cross-sectional area of the airway could be made (Fig. 3D). In response to MCh, the airway contracted to a smaller stable size and this correlated with the initiation and increase in the frequency of the Ca^2+ ^oscillations to a stable rate. Similarly, the ISO-induced decrease in the frequency of the Ca^2+ ^oscillations correlated with the ISO-induced initial relaxation of the airway. The subsequent re-contraction of the airway correlated with the late increase in the frequency of the Ca^2+ ^oscillations.

The effect of ISO on the frequency of the Ca^2+ ^oscillations was expressed as the ratio of the frequency of the Ca^2+ ^oscillations after ISO exposure (60 to 90 seconds) to the frequency of the Ca^2+ ^oscillations before ISO exposure. In the range from 1 nM to 10 μM, ISO induced a concentration-dependent decrease in the frequency ratio from 0.93 ± 0.03 (n = 5 slices from 3 mice) to 0.56 ± 0.03 (n = 6 slices from 4 mice). The decline in the frequency ratio was inversely proportional to the change in airway size (Fig. 3E).

### Relaxation of airways by repeated exposure to ISO

**Figure 4:**
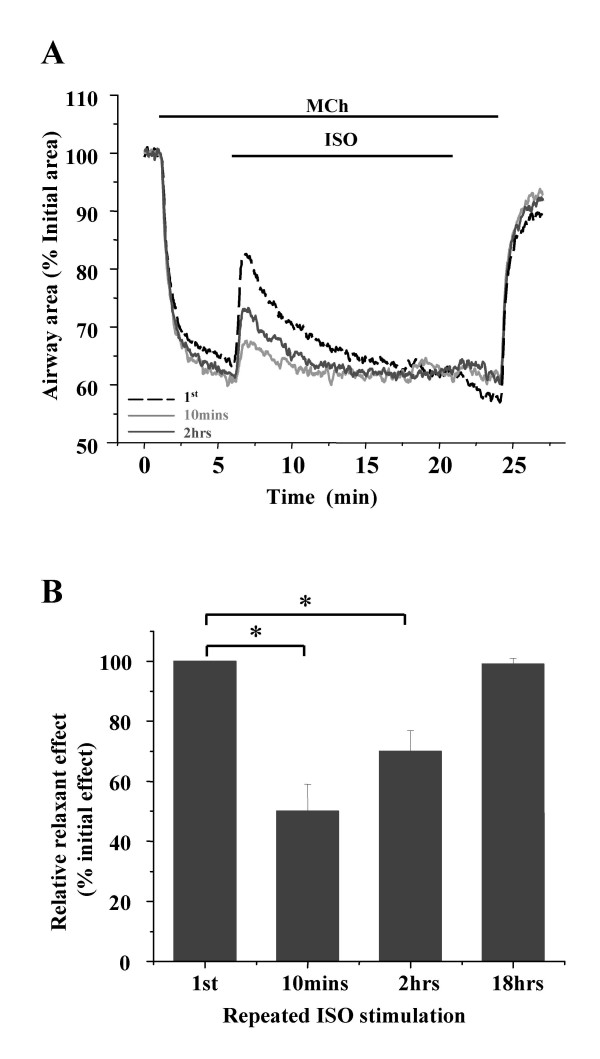
**The ability of ISO to relax an airway is influenced by repetitive stimulation**. **(A) **Relaxation responses of the same contacted airway (with MCh, 200 nM) in response to the first (black dash line) and subsequent 15 minute-exposures of ISO (1 μM) with the time intervals of 10 minutes (light grey line) and 2 hours (dark grey line). The slices were washed with sHBSS between experiments. **(B) **The summary of the relative relaxation responses induced by repeated ISO stimulation after different intervals. ISO-induced relaxation declined to about half after 10 minutes but was fully recovered after 18 hours. Relaxation was expressed as a percentage of the initial relaxation response. Data from experiments with 5 different airways from 4 mice (*, p < 0.05 compared to the 1^st ^relaxation).

Although we found that ISO induced a concentration-dependant relaxation of pre-contracted airway, this relaxation response did not persist in the presence of ISO. A possible explanation for this response is receptor accommodation or desensitization to the continual presence of agonists. To address this possibility, we examined the relaxation response of the same contracted airway (with MCh, 200 nM) to multiple exposures of ISO (1 μM) (Fig. 4). The interval between each ISO exposure was increased from 10 minutes to 2 hours and 18 hours. In comparison with the initial relaxation response to ISO, the subsequent relaxation of the airway decreased to 50 ± 9 % (n = 5 slices from 4 mice, p < 0.01) in response to ISO exposure after 10 minutes (Fig 4A and 4B). However, the efficacy of ISO was substantially improved when the interval between exposures was lengthened to 2 hours. After 18 hours, the ISO induced-relaxation was found to have recovered to 97 ± 3 % (n = 2 slices from 2 mice, P = 0.36 compared to initial effect) (Fig. 4A and 4B).

### Relaxing effect of FSK and 8-bromo-cAMP on contracted airways

**Figure 5:**
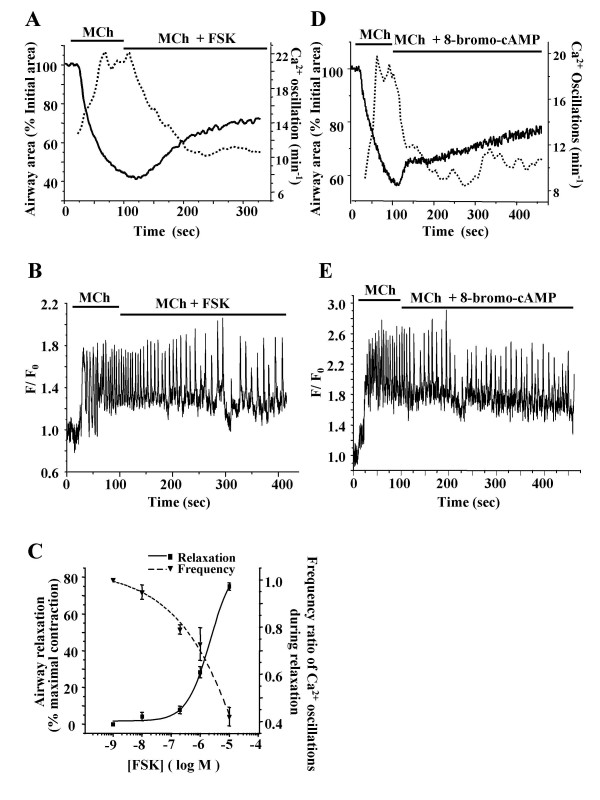
**The effect of FSK and 8-bromo-cAMP on contraction and Ca^2+ ^oscillations of airway SMCs. (A) **FSK (10 μM, solid line) and **(D) **8-bromo-cAMP (500 μM, solid line) induced an initial quick relaxation followed by slower relaxation. **(B) **FSK and **(E) **8-bromo-cAMP decreased the Ca^2+ ^oscillation frequency initiated by MCh to a slower rate. The changes in the frequency of the Ca^2+ ^oscillations induced by each compound (dotted line in **A **and **D**) correspond with opposite changes in airway area (solid line in **A **and **D**). (**C**) With increasing FSK concentration, the relaxation (solid line, ■) increased while the frequency of the Ca^2+ ^oscillations (dotted line, ▼) decreased; each point is the mean ± S.E. from at least 4 different airways from 3 mice. Data points were fitted with a logistic function.

To investigate the airway relaxation response to the elevation of [cAMP]_i_, we examined the effects of FSK, an activator of AC, and 8-bromo-cAMP, an active membrane permeable analogue of cAMP. Both FSK (10 μM) and 8-bromo-cAMP (500 μM) induced an initial fast relaxation followed by a slow progressive relaxation of MCh contracted airways (Fig. 5A and 5D solid line). The increase in the lumen area induced by 500 μM 8-bromo-cAMP was 49 ± 9 % (n = 3 slices from 2 mice). The effect of FSK was concentration-dependent (Fig. 5C); increasing concentrations of FSK (10 nM to 10 μM) induced an increasing relaxation (measured after 5 minutes exposure) from 4 ± 1 % to 75 ± 2 % (n = 4 slices from 3 mice).

Recordings of intracellular Ca^2+ ^in airway SMCs revealed that both FSK and 8-bromo-cAMP reduced the frequency of the Ca^2+ ^oscillations initiated by MCh (Fig. 5B and 5E). In contrast to the effects of ISO, the frequency of the Ca^2+ ^oscillations declined throughout the experiment. In range from 10 nM to 10 μM, FSK induced a concentration-dependent decrease in the frequency of the Ca^2+ ^oscillations (Fig. 5C). The frequency of the Ca^2+ ^oscillations after 5 minutes of FSK exposure, expressed as the ratio to the Ca^2+ ^oscillation frequency induced by MCh exposure, decreased from 0.95 ± 0.03 to 0.42 ± 0.04 (n = 4 slices from 3 mice) as the FSK concentration increased. The changes in frequency of the Ca^2+ ^oscillations induced by FSK or 8-bromo-cAMP correlated with a progressive relaxation of the airway (Fig. 5A and 5D).

### A decrease in Ca^2+ ^oscillation frequency correlates with airway relaxation

**Figure 6:**
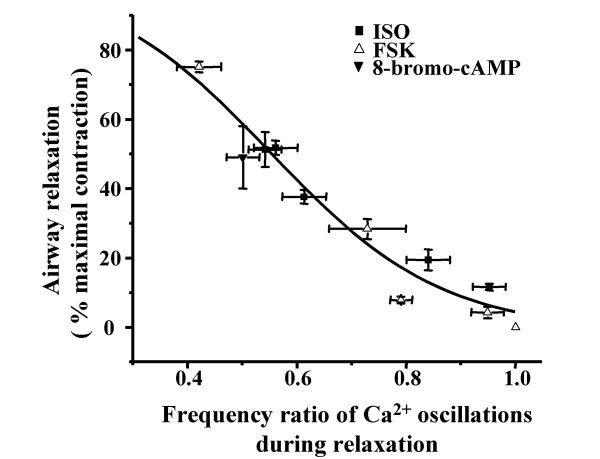
**Relationship between the reduction in Ca^2+ ^oscillation frequency in SMCs and airway relaxation. **The relaxation state (percentage of the MCh-induced contraction) and corresponding Ca^2+ ^oscillation frequency (ratio to the frequency of MCh-induced Ca^2+ ^oscillations) upon exposure to ISO, FSK and 8-bromo-cAMP (at various concentrations) were plotted and fitted with a logistic function. The data indicates that a greater airway relaxation correlated with a larger reduction in the Ca^2+ ^oscillation frequency.

During all of the relaxation responses to ISO, FSK or 8-bromo-cAMP, the frequency of the Ca^2+ ^oscillations initiated by MCh was reduced (Fig. 3D, Fig. 5A and 5D). A summary plot of these data (from Fig. 3E, Fig. 5C and 5D) is presented in figure 6. This analysis suggests that, irrespective of the agonist, the frequency of the Ca^2+ ^oscillations determines the extent of airway relaxation: the lower the frequency of the Ca^2+ ^oscillations in airway SMCs, the greater the relaxation of the airway (Fig. 6).

### cAMP does not affect caffeine-induced Ca^2+ ^release

**Figure 7:**
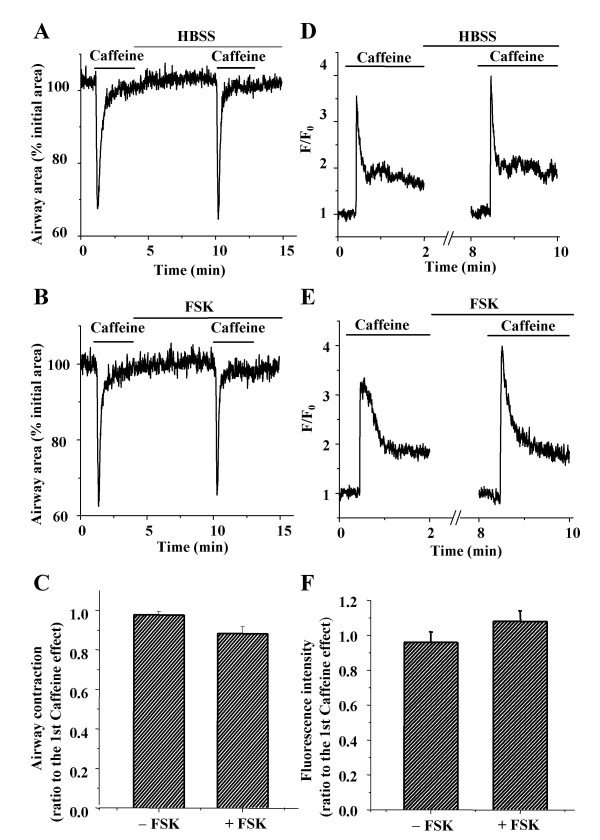
**Effect of FSK on caffeine-induced Ca^2+ ^release. **Representative experiments demonstrating **(A) **contraction and **(D) **Ca^2+ ^signaling of airway SMCs induced by repetitive exposure to caffeine (20 mM) in the absence of FSK. FSK (10 μM) had no effect on caffeine-induced **(B) **contraction or **(E) **Ca^2+ ^signaling. **(C) **Airway contraction and **(F) **the peak fluorescence intensity of Ca^2+ ^signaling (ratio of second to first exposure) induced by caffeine (20 mM) in the slices incubated with or without FSK (10 μM). Data represents the mean ± S.E. from 4 different slices from 3 mice.

To explore the mechanisms by which cAMP reduced the frequency of the Ca^2+ ^oscillations, we investigated the effect of cAMP on caffeine-induced Ca^2+ ^release and refilling of intracellular Ca^2+ ^stores (Fig. 7). In view of the transient nature of ISO-induced relaxation, FSK was used to elevate cAMP because of its direct and stable action on AC. Upon exposure to 20 mM caffeine, the airways responded with a transient contraction (Fig 7A). This treatment also induced in the SMCs a transient increase in [Ca^2+^]_i _(peak fluorescence intensity ratio, F/F_0 _= ~3.5) followed by a sustained plateau of [Ca^2+^]_i _(fluorescence ratio = ~1.8, Fig. 7D). The incubation of the same airway with FSK (10 μM for 5 min) had no significant effect on caffeine-induced airway contraction (Fig. 7B) or [Ca^2+^]_i _elevation (Fig. 7E). The ratio of contraction induced by the second exposure to caffeine as compared to the first exposure was 0.96 ± 0.02 in the absence of FSK and 0.88 ± 0.04 in the presence of FSK (Fig. 7C). The ratios of the peak fluorescence intensities were 0.96± 0.06 in the absence of FSK and 1.0 ± 0.06 in the presence of FSK (n = 4 slices from 3 mice, Fig. 7F). The similarity of the results suggests that cAMP does not affect caffeine-induced Ca^2+ ^release (i.e. via the ryanodine receptor) from the SR or the subsequent refilling of the depleted internal Ca^2+ ^store.

### Supplementation of intracellular Ca^2+ ^does not block the relaxing effect of cAMP

Because a reduction of [Ca^2+^]_i_, brought about by decreasing Ca^2+ ^influx and/or increasing Ca^2+ ^efflux or reuptake, has been proposed as a mechanism by which cAMP relaxed airway SMCs, we examined the effect of elevating [Ca^2+^]_i _during the response of SMCs to cAMP. The [Ca^2+^]_i _was increased with 3 different agents: 1) NP-EGTA, a caged-Ca^2+ ^compound, which upon UV photolysis releases Ca^2+^, 2) ionomycin, a Ca^2+ ^selective ionophore, and 3) iberiotoxin (IbTX), a specific inhibitor of BK_Ca _channels, which enhances Ca^2+ ^influx by blocking K^+ ^efflux to induce hyper-polarization.

**Figure 8:**
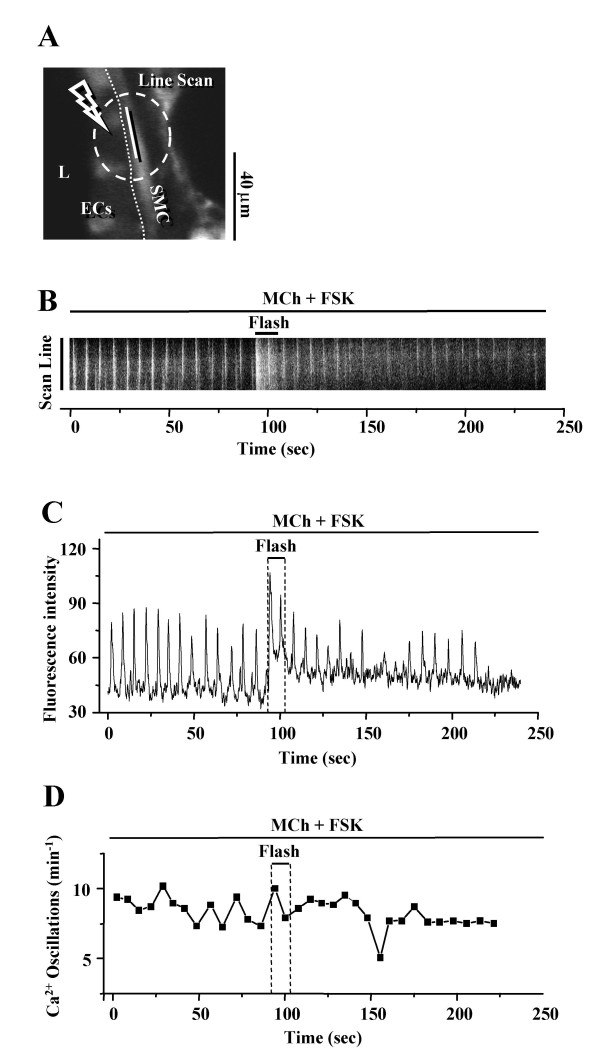
**Effect of flash photolysis of caged-Ca^2+ ^on SMCs treated with MCh and FSK. (A) **A fluorescence image of part of an airway obtained by confocal microscopy. L, lumen; ECs, epithelial cells; SMC, smooth muscle cell. The dashed white oval indicates the position and size of the zone of UV illumination (10 s, OD 0.6). Dotted line indicates the interface between ECs and SMCs. Scale bar = 40 μm. The intracellular Ca^2+ ^signaling represented by**(B) **a line-scan plot, constructed by sequentially aligning the pixels along the length of the SMC (white line indicated in **A**) and from each recorded fluorescence image and **(C) **a selected ROI within the SMC and **(D) **the frequency of Ca^2+ ^oscillations of SMCs in response to flash photolysis of caged-Ca^2+ ^during stimulation with MCh (200 nM) and FSK (10 μM). Flash photolysis induced a temporary rise in [Ca^2+^]_i _but did not increase the slow frequency of the Ca^2+ ^oscillations. Representative traces of 6 different slices from 4 mice.

In airway SMCs pre-treated with MCh (200 nM) and FSK (10 μM), flash photolysis of caged-Ca^2+ ^within the SMC increased the fluorescence intensity of intracellular Ca^2+ ^signaling (Fig. 8B). However this served only to elevate the baseline, but not the frequency, of the Ca^2+ ^oscillations (Fig. 8C and 8D, 6 airways from 4 mice). No change in the airway luminal area or an increase in contraction of the illuminated SMCs was observed.

**Figure 9:**
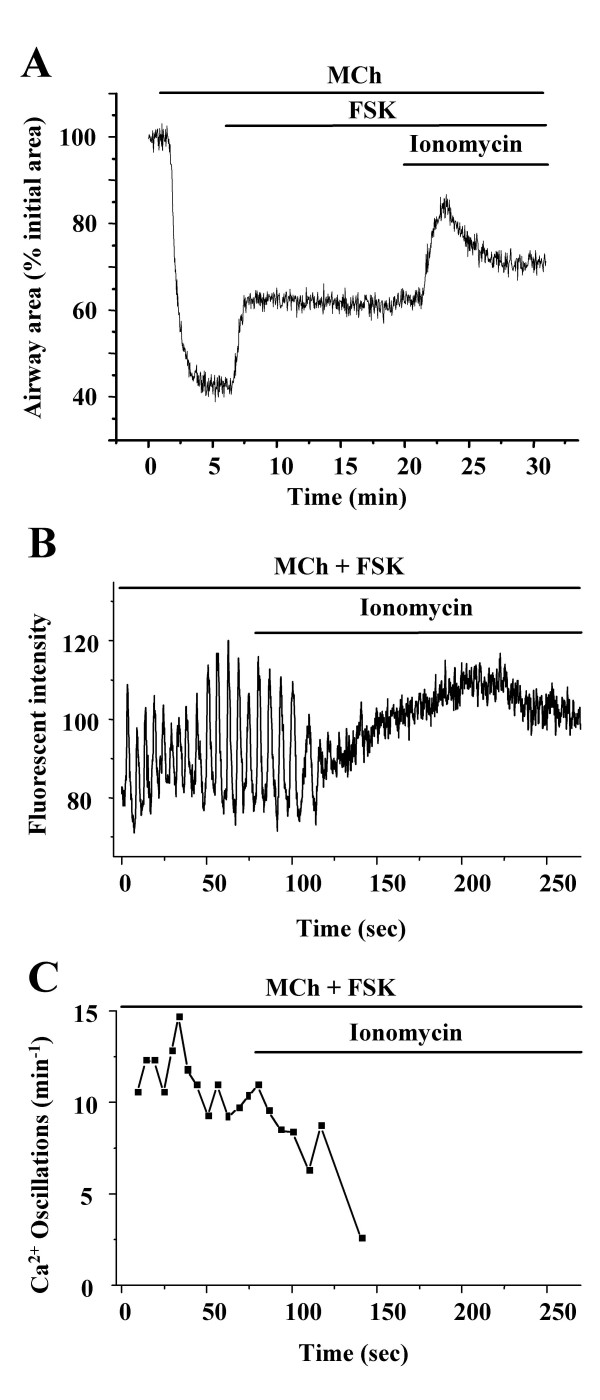
**Effect of ionomycin on FSK-induced airway relaxation. (A) **Airway contraction in response to MCh (200 nM), FSK (10 μM) and ionomycin (5 μM). Ionomycin initially enhanced the relaxation induced by FSK but subsequently re-contracted the airway. Representative traces from 4 experiments from 3 mice. (**B) **Changes in intracellular Ca^2+ ^and **(C) **the frequency of the Ca^2+ ^oscillations induced by MCh (200 nM) and FSK (10 μM) followed by exposure to ionomycin. Ionomycin stopped the Ca^2+ ^oscillation and subsequently elevated the [Ca^2+^]_i_. Representative traces of five experiments from 4 mice.

Upon exposure to ionomycin (5 μM), the relaxation induced by FSK (10 μM) in MCh (200 nM) contracted airway SMCs was initially enhanced, reaching a maximal relaxation after 2 min (Fig. 9A). This additional relaxation correlated with the cessation of the Ca^2+ ^oscillations (Fig. 9B and 9C). After continued ionomycin exposure, the airway began to re-contract and this correlated with an steady increase in the [Ca^2+^]_i _(Fig 9A and 9B) (5 airways from 4 mice).

**Figure 10:**
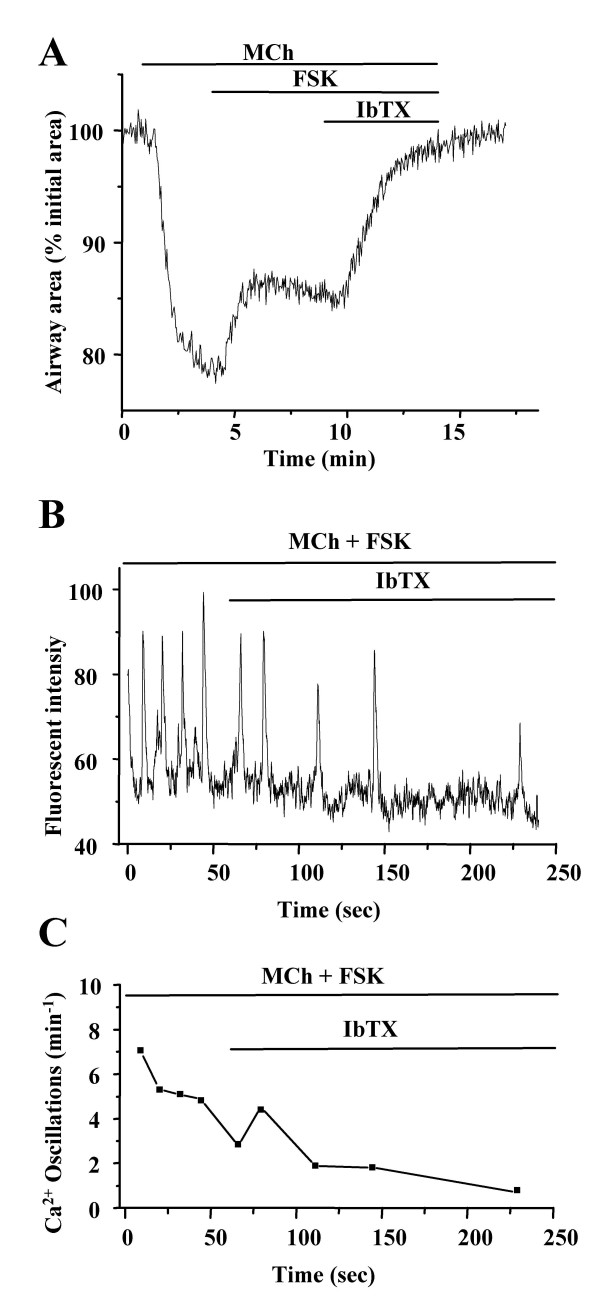
**Effect of iberiotoxin on FSK-induced airway relaxation. (A) **Airway contraction in response to MCh (200 nM), FSK (10 μM) and IbTX (50 nM). IbTX greatly enhanced the relaxation induced by FSK to fully relax the airway. Representative traces of 6 experiments from 3 mice. (**B) **Changes in intracellular Ca^2+ ^signaling and **(C) **the frequency of Ca^2+ ^oscillations induced by MCh (200 nM) and FSK (10 μM) followed by exposure to IbTX (50 nM). The frequency of the Ca^2+ ^oscillations was further slowed by IbTX. Representative traces of 8 experiments from 5 mice.

A similar change in the contraction and [Ca^2+^]_i _of airway SMCs was observed when the lung slices treated with MCh (200 nM) and FSK (10 μM) were subsequently exposed to IbTX (50 nM). The airway responded with a quick and full relaxation within 3 mins (Fig. 10A). Again, the accompanying change in the [Ca^2+^]_i _revealed a rapid slowing of the frequency of the Ca^2+ ^oscillations to a full stop. In contrast to the effect of ionomycin, the [Ca^2+^]_i _of SMCs remained near baseline after the Ca^2+ ^oscillations ceased (Fig. 10B, 6 airways from 5 mice).

In summary, we used three different methods to increase the [Ca^2+^]_i _during FSK- induced relaxation but the frequency of the Ca^2+ ^oscillation could not be increased to reverse the FSK effect. Moreover, the increase of [Ca^2+^]_i _induced by ionomycin inhibited the Ca^2+ ^oscillations to induce further relaxation. These data suggest that a reduction in the availability of Ca^2+ ^is not the cause of cAMP-induced slowing of Ca^2+ ^oscillations.

### cAMP exerts a relaxant effect by inhibiting IP3-induced Ca^2+ ^release

Because Ca^2+ ^release through the IP_3 _receptor of internal Ca^2+ ^stores is a major mechanism contributing to the formation of Ca^2+ ^oscillations, we investigated whether cAMP inhibited IP_3_-induced Ca^2+ ^release. When airway SMCs loaded with caged-IP_3 _were illuminated with UV light for about 1 s, several Ca^2+ ^oscillations were observed to propagate as Ca^2+ ^waves from the illuminated zone through the rest of the SMC (data not shown). When the intensity and exposure time of UV light was optimized, repetitive UV flashes could induce similar repetitive Ca^2+ ^waves in the same SMC.

**Figure 11:**
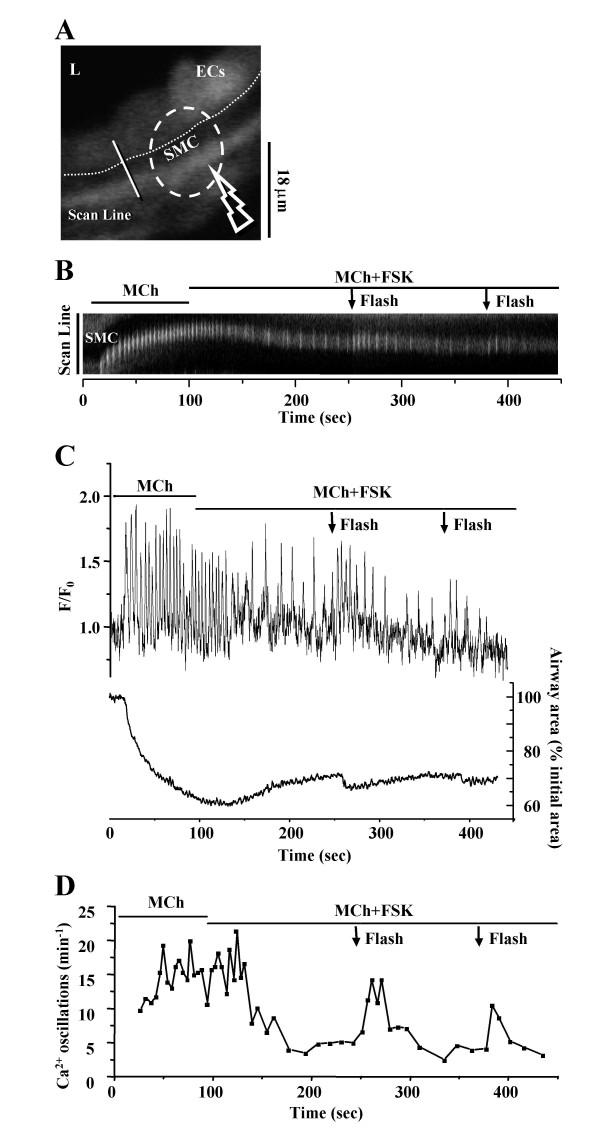
**Effect of flash photolysis of caged-IP_3 _on SMCs treated with MCh and FSK. (A) **A fluorescence image of part of an airway obtained by confocal microscopy. The dashed white oval indicates the position and size of the zone of UV illumination (0.5 s, OD 0.5). L, lumen; ECs, epithelial cells; SMC, smooth muscle cell. Dotted line indicates the interface between ECs and SMCs. Scale bar = 18 μm. Intracellular Ca^2+ ^signaling represented by **(B) **a line-scan plot, constructed by sequentially aligning the pixels along the line across the SMCs and ECs (white line indicated in **A) **from each recorded fluorescence image. **(C) **The simultaneous recording of intracellular Ca^2+ ^signaling of SMC (upper trace, and **B**), and the changes in airway lumen area (lower trace) and **(D) **frequency of the Ca^2+ ^oscillations in response to the flash photolysis of caged-IP_3 _during stimulation with MCh (200 nM) and FSK (10 μM). Repetitive release of IP_3 _by flash photolysis (0.5 s, OD 0.5, indicated by arrows) increased the frequency of the Ca^2+ ^oscillations induced by MCh and FSK and re-contracted the airway. Representative traces of 6 different slices from 4 mice.

A similar set of experiments was then performed in the presence of MCh and FSK. The inhibition of the frequency of the Ca^2+ ^oscillations and the accompanying relaxation of SMCs were observed when the slices were exposed to MCh (200 nM) and FSK (10 μM). But, in response to UV illumination (0.5s, OD 0.5) and photolysis of caged IP_3_, the frequency of the Ca^2+ ^oscillations increased significantly for a short period (Fig. 11B, C, D). A second flash again increased the oscillation frequency, although to a lesser extent. Simultaneous recording of changes in the frequency of Ca^2+ ^oscillations and contraction of airway SMCs revealed that the airway re-contracted after each UV flash (Fig. 11C). These results suggest that FSK exerts its relaxant effect by inhibiting the IP_3 _induced Ca^2+ ^release from internal Ca^2+ ^stores.

**Figure 12:**
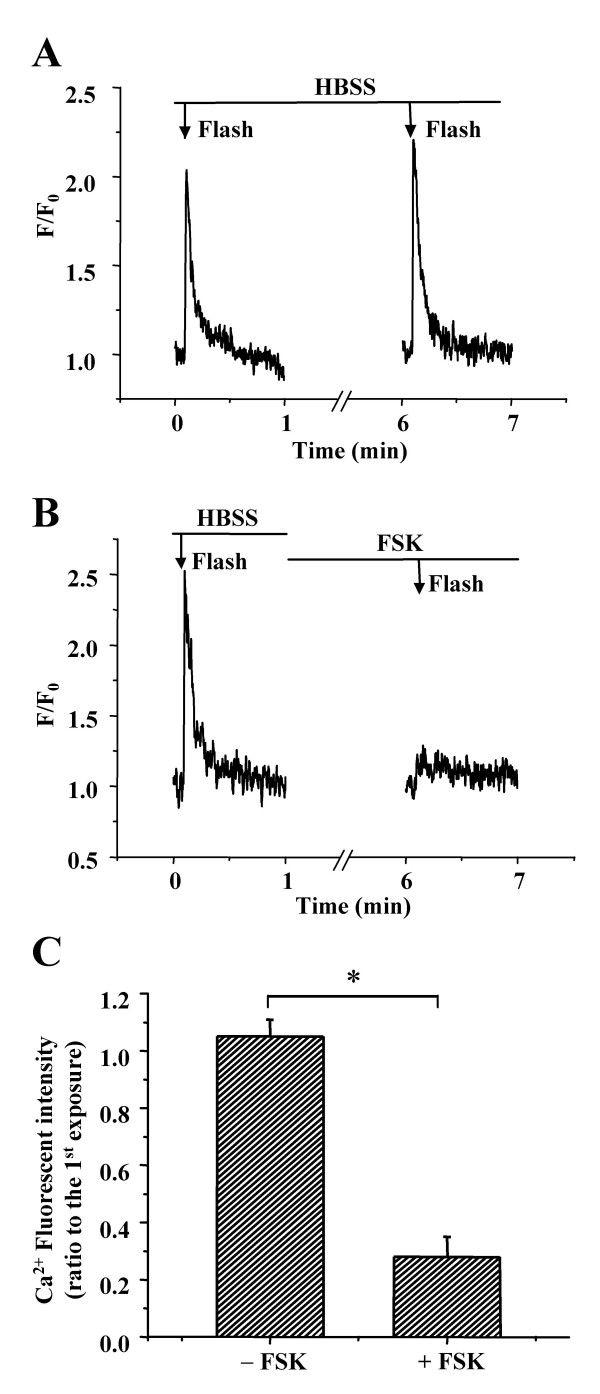
**Effect of FSK on the intracellular Ca^2+ ^signaling induced by IP**_3_. The Ca^2+ ^signaling of SMCs induced by repetitive flash-photolysis (0.5 s, OD 1.0, arrows) of caged-IP_3 _in lung slices incubated **(A) **without or **(B) **with FSK for 5 min. Exposure to FSK almost completely abolished the Ca^2+ ^response induced by flash photolysis of IP_3_. **(C) **Summary of the changes in Ca^2+ ^signaling (peak fluorescence intensity) induced by IP_3 _in the slices incubated without or with FSK. The Ca^2+ ^signal after exposure to the sHBSS without or with FSK was expressed as a ratio to the Ca^2+ ^response before exposure. Data represents the mean ± S.E. from at least 5 different slices from 4 mice. *, p < 0.05.

If the IP_3 _receptor is inhibited by cAMP, it should be possible to block Ca^2+ ^release induced by IP_3 _in cAMP treated SMCs. In control experiments, a lower level of UV illumination (reduced to 1/3, by using a neutral density filter with OD 1.0) induced a single spike of elevated [Ca^2+^]_i _in untreated airway SMCs (Fig. 12). A second flash gave a similar response. However, after the SMCs were treated with FSK (10 μM) for 5 minutes, the increase in [Ca^2+^]_i _was virtually abolished in response to the UV flash (Fig. 12B and 12C).

### CICR through the ryanodine receptor is not required for Ca^2+ ^oscillations

**Figure 13:**
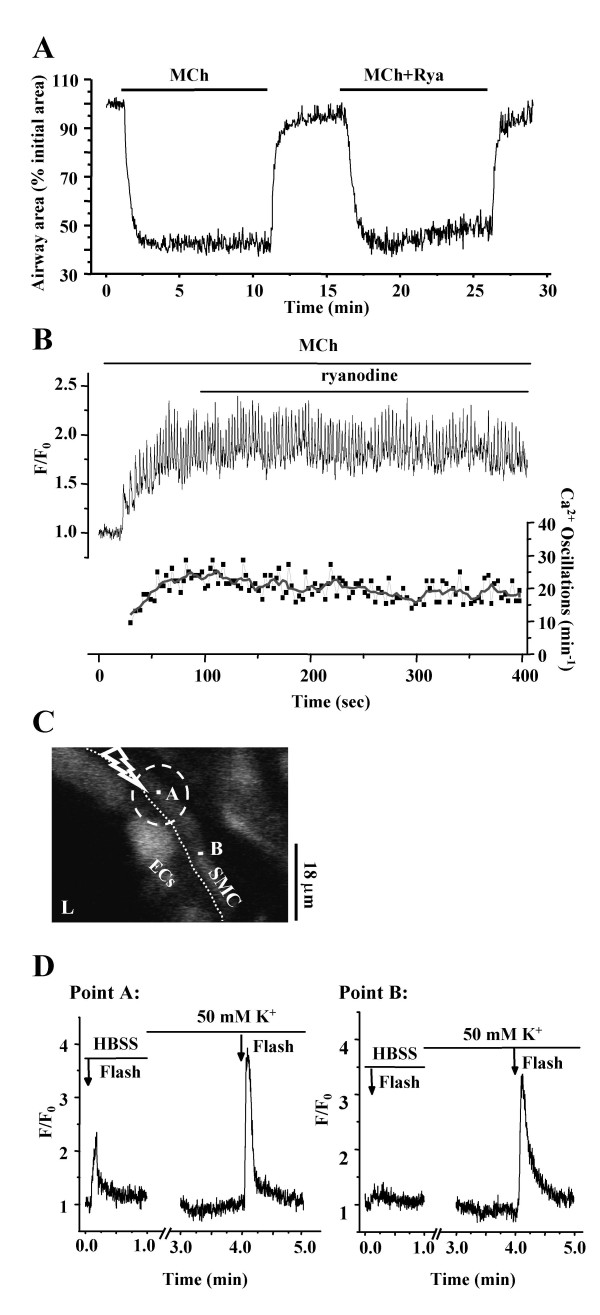
**Effect of the ryanodine on contraction and Ca^2+ ^signaling. (A) **MCh (200 nM) induced contraction of the same airway in the absence or presence of ryanodine (50 μM, Rya). **(B) **Changes in fluorescence (upper trace) and frequency of oscillatory Ca^2+ ^signaling (lower trace) of a single airway SMC treated with 200 nM MCh and 50 μM ryanodine. Representative traces of 6 different slices from 4 mice.**(C) **A fluorescence image of part of the airway obtained by confocal microscopy. The dashed white oval indicates the position and size of the zone of UV illumination (5 s, OD 0.6). L, lumen; ECs, epithelial cells; SMC, smooth muscle cell. Dotted line indicates the interface between ECs and SMCs. Scale bar = 18 μm. Point A was located at the center of the zone of illumination on a single SMC and Point B was located on the same SMC outside the zone of illumination. **(D) **The local Ca^2+ ^signals at point A and B induced by flash photolysis of caged-Ca^2+ ^in the absence or presence of 50 mM K^+ ^(incubation time of 3 min). Representative traces of 5 different slices from 3 mice.

Because the ryanodine receptor (RyR) is involved in Ca^2+^-induced Ca^2+ ^release (CICR) in a variety of cell types, we examined the effect of CICR via RyR in airway SMCs. We found that exposure to ryanodine (50 μM, sufficient to inhibit the RyR) induced only a small and slow decrease in the MCh-induced airway contraction (~5%, after 5 minutes, Fig. 13A) and frequency of Ca^2+ ^oscillation in the SMCs (~4 min^-1^, after 5 minutes, Fig. 13B). The amplitude of the Ca^2+ ^oscillations was unaffected. These result suggest that the increases in the [Ca^2+^]_i _associated with the Ca^2+ ^oscillations are not mediated by RyR or are sufficient to invoke CICR via the RyR.

To examine the Ca^2+ ^conditions required for the induction of CICR, we released Ca^2+ ^by flash photolysis of caged Ca^2+^. When a restricted area within a single SMC was exposed to UV light (Fig 13C), only a brief localized increase in [Ca^2+^]_i _was detected (Fig. 13D). The increase in Ca^2+^, as determined by the fluorescence change, was similar to that induced by a Ca^2+ ^oscillation, but this Ca^2+ ^change did not spread to the rest of the cell. By contrast, when the same airway SMCs was treated with sHBSS containing 50 mM K^+ ^for ~3 minutes, a second identical UV flash induced a larger Ca^2+ ^release in the zone of illumination and induced a Ca^2+ ^wave that propagated throughout the cell (Fig 13D, 5 airways from 3 mice). Treatment with high K^+ ^was used because it has previously been reported that high K^+ ^initiates membrane-depolarization that results in excessive Ca^2+ ^influx, overfilling of internal Ca^2+ ^store and sensitization of the RyR [14]. These results indicate that CICR through RyR only occurs in airway SMCs under certain circumstances or "stressed conditions" and implies that RyRs do not play a significant role in the normal maintenance of agonist-induced Ca^2+ ^oscillations.

## Discussion

In this study, thin lung slices were used to explore the changes in intracellular Ca^2+ ^that occur in airway SMCs during relaxation induced by ISO and other cAMP elevating compounds. The airway SMCs were initially contracted with MCh, an analogue of ACh, which is more slowly metabolized and widely used in clinical lung function tests. We found that MCh induced both a concentration-dependent contraction of the airways and increase in the frequency of Ca^2+ ^oscillations within airway SMCs. These results are similar to our previous findings obtained with ACh in lung slices [12,14]. Upon exposure to ISO, the MCh-contracted airways initially displayed a transient relaxation but subsequently re-contracted. The ability of ISO to relax the airway was dependent both on the ISO concentration and the MCh concentration used to contract the airway. If the airways were contracted with high concentration of MCh (1 μM), ISO, even at high concentrations (i.e.10 μM) was ineffective at relaxing the airway. Only at low MCh concentrations was ISO able to induce substantial relaxation. A similar relaxation and subsequent re-contraction in response to ISO (10 nM ~10 μM) was also observed in MCh-contracted (1 μM) rat lung slices (data not shown). These results suggest that the relaxing action of ISO is relatively weak in comparison to the contractile action of MCh and that the airway becomes de-sensitized to ISO-induced relaxation to allow re-contraction.

In addition to being activated by β-adrenoceptor associated G-stimulatory proteins (G_s_), AC can also be inhibited by receptor-coupled G-inhibitory proteins (G_i_). Therefore, a potential mechanism for the down-regulation of the ISO response is the inhibition of AC by activation of M_2 _muscarinic receptors and their associated G_i _by MCh [28,29]. However, in the presence of the M_2 _receptor antagonist, methoctramine, MCh only induced a slightly smaller airway contraction (about 95%), but more importantly, ISO induced a similar transient relaxation. This transient response to a high concentration of ISO also persisted across a wide range of MCh concentrations, which would be expected to differentially activate the M_2 _receptors. Moreover, a similar transient response to ISO was also observed in the airways contracted by 5-HT, an agonist with no effect on the AC. In other studies [30,31], the pretreatment of cultured SMCs or bronchial smooth muscle strips with ISO (30 min to 24 hours) reduced subsequent ISO-mediated cAMP generation and relaxation. Similarly, we found that repetitive exposure of the airway to ISO (1 μM, 10 min to 2 hours) significantly decreased the relaxation response (by 30 to 50%). These results indicate that the M_2 _receptor has a minimal effect on AC activity at mid-range MCh concentrations and that the down-regulation of the ISO response is mediated by rapid de-sensitization and slow re-sensitization at the β-adrenoceptor. Desensitization of the β-adrenoceptor can be mediated by phosphorylation of the receptor by cAMP-independent β-adrenoceptor kinase or other G protein-coupled receptor kinases or by receptor internalization – a process that takes a longer time to reverse (hours) [2,32].

Because β-adrenoceptor agonists primarily relax SMCs by increasing [cAMP]_i_, an implication of receptor desensitization is that the [cAMP]_i _within the SMCs initially increases but subsequently declines. The idea that increases in [cAMP]_i _correlate with relaxation was addressed by circumventing the β-adrenergic receptor by either activating adenylyl cyclase with FSK or by increasing the [cAMP]_i _directly with 8-bromo-cAMP. A major characteristic of FSK or 8-bromo-cAMP-induced relaxation was that the relaxation was sustained and that re-contraction never occurred. This response is consistent with the idea that the [cAMP]_i _must be maintained or increased if relaxation is to be sustained and that some form of desensitization of β-adrenoceptor occurs allowing re-contraction.

The correlation of increases in [cAMP]_i _with airway relaxation has been reported in mice [33], rats [23,34], guinea pigs [35], rabbits [36,37], dogs [3], cows [4,24,38], and humans [24,37]. However, these studies were all conducted on isolated airway muscle strips or tracheal rings by measuring changes in isometric tension. Consequently, to explore the cellular mechanisms by which ISO and other agents acting via cAMP to relax SMCs, we examined their effects on intracellular Ca^2+^. As previously described, MCh, as well as ACh, initiates Ca^2+ ^oscillations in SMCs and the frequency of these Ca^2+ ^oscillations determines the extent of the SMC contraction [9,11,12,14]. During the initial response to ISO, the frequency of the MCh-induced Ca^2+ ^oscillations was quickly reduced and this was accompanied by fast relaxation of the airway. However, after a short time, the Ca^2+ ^oscillation frequency began to slowly increase and this was accompanied by the slow re-contraction of the airway. A similar slowing of the frequency of the Ca^2+ ^oscillations and airway relaxation was induced by FSK and 8-bromo-cAMP. But, in contrast to ISO, the frequency of the Ca^2+ ^oscillations continued to slow to lower frequencies; a result that correlates with the greater airway relaxation. Although the relationship to contraction could not be measured, Salbutamol, a β_2_-adrenoceptor agonist, has also been found to slow and reduce the baseline of ACh-induced Ca^2+ ^oscillation in dissociated porcine tracheal SMCs [16]. In our studies, we found that the baseline of the Ca^2+ ^oscillations was relatively stable. Consequently, we hypothesized, especially in view of the fact that the peak [Ca^2+^]_i _of the Ca^2+ ^oscillations is substantially high, that increases in [cAMP]_i _induce airway relaxation by reducing the frequency of Ca^2+ ^oscillations in SMCs rather than by lowering the baseline of global [Ca^2+^]_i_.

We [12-14] and others [10,39] have shown that agonist-induced Ca^2+ ^oscillations in airway SMCs are generated by cycles of Ca^2+ ^release and uptake by the sarcoplasmic reticulum (SR). Because extracellular Ca^2+ ^is required to replenish internal stores, it has been proposed that cAMP might exert its action on Ca^2+ ^oscillations by 1) the inhibition of Ca^2+ ^replenishment of the internal stores by inhibiting Ca^2+ ^pumps and/or 2) blocking the Ca^2+ ^influx through voltage-dependent Ca^2+ ^channels (VOC) or store-operated Ca^2+ ^channels (SOCC), or 3) by inhibiting the Ca^2+ ^release via IP_3 _and/or ryanodine receptors.

To test the first hypothesis, that the internal Ca^2+ ^stores were depleted, we investigated the effects of caffeine on cAMP-treated lung slices. We found that elevated [cAMP]_i _did not significantly affect the Ca^2+ ^signaling or contractile response induced by repetitive exposures to caffeine. These results demonstrate that the Ca^2+ ^capacity of the internal store was fully restored to normal in the presence of cAMP and imply that Ca^2+ ^loading of the SR by Ca^2+ ^pumps was not significantly altered.

To investigate the second possibility that cAMP decreased Ca^2+ ^influx to limit the Ca^2+ ^available to replenish internal stores and maintain the ongoing Ca^2+ ^oscillation, we used flash photolysis of caged-Ca^2+ ^or exposure to ionomycin, a Ca^2+ ^ionophore, to directly increase the available Ca^2+^. We found that while a transient global increase in [Ca^2+^]_i _resulted from the photolysis of caged-Ca^2+^, this had little effect on the slow frequency of the Ca^2+ ^oscillations in the presence of FSK. Furthermore, the application of ionomycin initially inhibited the FSK-reduced Ca^2+ ^oscillation to relax the airway. However, the gradual increase in [Ca^2+^]_i _induced by continued exposure to ionomycin subsequently induced re-contraction of the airway. Because in both experiments, an increase in the frequency of the Ca^2+ ^oscillations was not observed, these results indicate that a lowered Ca^2+ ^influx is not a limiting factor. It is possible that ionomycin might have emptied the Ca^2+ ^of SR to inhibit the Ca^2+ ^oscillations; however considering the higher concentration of Ca^2+ ^in the extracellular environment it is unlikely that the internal store would have been emptied before an influx of Ca^2+ ^had occurred.

Another mechanism inhibiting SMC Ca^2+ ^influx appears to involve membrane hyper-polarization resulting from cAMP-activation of BK_Ca _channels to down-regulate voltage-dependent Ca^2+ ^influx [19-21]. Specific blockers of the BK_Ca _channels such as IbTX or charybdotoxin have been shown to antagonize cAMP-induced relaxation in isolated trachea or bronchi from guinea-pigs [21], pig [40], horse [41]. However, in keeping with the effects of ionomycin, we found that in lung slices treated with MCh and FSK, IbTX further inhibited the Ca^2+ ^oscillations to enhance airway relaxation. This effect suggests IbTX (and ionomycin) may actually inhibit Ca^2+ ^oscillation by enhancing Ca^2+ ^influx. The failure to observe a significant elevation of baseline Ca^2+ ^suggests either that the influx of Ca^2+ ^is sufficiently low and quickly sequestered into internal stores or that an alternative, non-specific action of IbTX must be considered. The failure of IbTX to induce re-contraction is consistent with the fact that voltage-dependent Ca^2+ ^influx does not appear to play a prominent role in maintaining the agonist-induced Ca^2+ ^oscillations in airway SMCs and that airway contraction and Ca^2+^signaling induced by membrane depolarization (with KCl) were completely different to that induced by agonists [42]. Furthermore, there are conflicting reports regarding the contribution of BK_Ca _channels based on differences amongst species and regions of the airway as well as experimental conditions [43,44]. Consequently, it appears unlikely that the BK_Ca _channel is a major mechanism mediating the effects of cAMP in mouse airway SMCs. Taken together, these data led us to believe that decreased [Ca^2+^]_i _is not the primary cause of the modulation of the frequency of Ca^2+ ^oscillations.

Therefore, we examined the third idea that cAMP altered the Ca^2+ ^release from SR. Although the RyR has an important role in CICR in many other cell types [45], Ca^2+ ^oscillations and airway contraction induced by MCh were minimally affected in the short term by the presence of ryanodine. This suggests that the Ca^2+ ^oscillations do not require RyRs and therefore it is unlikely that any effects of cAMP on RyR will be significant. The slight decline in the Ca^2+ ^oscillation frequency may be explained by an increased leak of Ca^2+ ^through RyR locked in the open state by ryanodine. However, this process must be occurring independently of the Ca^2+ ^oscillations otherwise the Ca^2+ ^oscillations would quickly stop as a result of empty Ca^2+ ^stores. These results are also consistent with the fact that the release of caged-Ca^2+ ^only initiated CICR when the cell was previously depolarized with KCl to overload the internal Ca^2+ ^stores and sensitize the RyR.

The IP_3_R is another important Ca^2+ ^release channel of the SR and we found that the slow frequency of Ca^2+ ^oscillations induced by FSK could be increased when the [IP_3_]_i _was increased by flash photolysis. This response was accompanied by the re-contraction of airway SMCs. The idea that the sensitivity of the IP_3_R to IP_3 _is down-regulated by FSK is further supported by the finding that photolysis of caged IP_3 _induced a propagating Ca^2+ ^wave in the absence, but not in the presence of FSK. A similar effect of cAMP on Ca^2+ ^release has also been reported in other types of SMCs or non-excitable cells [46-49]. The underlying mechanism may involve cAMP-dependent PKA to phosphorylate the IP_3_R. However, the outcome of phosphorylation differs with the preparation and either an enhancement or inhibition of Ca^2+ ^release can result [46-49]. Alternatively, cAMP may influence the IP_3_R via EPACs.

Although the molecular details mediating the action of cAMP will require further investigation, we propose that the cAMP has an inhibitory effect on the IP_3_R and reduces the open probability of the receptor. Moreover, cAMP may also change the Ca^2+ ^sensitivity of the IP_3_R. Normally, the open probability of the IP_3_R is a bell-shaped function related to the [Ca^2+^]_i_; the receptor is closed by high Ca^2+ ^concentrations at a constant IP_3 _concentration [50]. With a decrease in [IP_3_]_i_, the bell-shaped response curve is shifted to the left, with the result that the IP_3_R closes at a lower [Ca^2+^]_i _[51,52]. In view of this change in sensitivity, we hypothesize that the inhibition of IP_3_R by cAMP results in a similar leftward and downward shift of the bell-shaped curve. This idea is consistent with our observation that in the presence of cAMP, an increase in Ca^2+ ^influx, induced by IbTX or ionomycin, leads to the closure of the IP_3_R to mediate the cessation of Ca^2+ ^oscillations and relaxation of the airway SMCs.

An additional mechanism that would augment the inhibitory action of cAMP on the IP_3_R is a reduction in the production of IP_3_. This idea is based on the evidence that G protein-activated PLCβ activity can be inhibited by PKA-induced phosphorylation [4,5,53,54]. This requires further investigation but is beyond the scope of this study.

An alternative mechanism for cAMP-induced SMC relaxation is the desensitization of contractile apparatus to Ca^2+ ^[8,24,40,55]. Phosphorylation of MLCK can lead to decreased affinity of MLCK for Ca^2+^/calmodulin while phosphorylation of Rho-kinase or protein kinase C can increase the activity of MLCP. We have also found that cAMP can down-regulate the Ca^2+^-sensitivity of agonist-induced contraction in lung slices [56]. However, it is not yet clear if such changes occur in parallel with the changes in Ca^2+ ^oscillation frequency.

In summary, we conclude that the frequency of Ca^2+ ^oscillations controls the contractile state of airway SMCs and that compounds that elevate cAMP slow the frequency of Ca^2+ ^oscillations within SMCs to proportionally induce airway relaxation. Furthermore, cAMP appears to regulate the Ca^2+ ^oscillation frequency by inhibiting Ca^2+ ^release through IP_3_R rather than influencing Ca^2+ ^influx or the refilling of internal stores. In addition, we find that, under normal conditions, the RyR is not essential for Ca^2+ ^oscillations of airway SMCs.
